# Comparative Study: Impacts of Ca and Mg Salts on Iron Oxygen Carriers in Chemical Looping Combustion of Biomass

**DOI:** 10.1021/acsomega.1c02138

**Published:** 2021-06-16

**Authors:** Duygu Yilmaz, Britt-Marie Steenari, Henrik Leion

**Affiliations:** Chemistry and Chemical Engineering, Chalmers University of Technology, 412 96 Gothenburg, Sweden

## Abstract

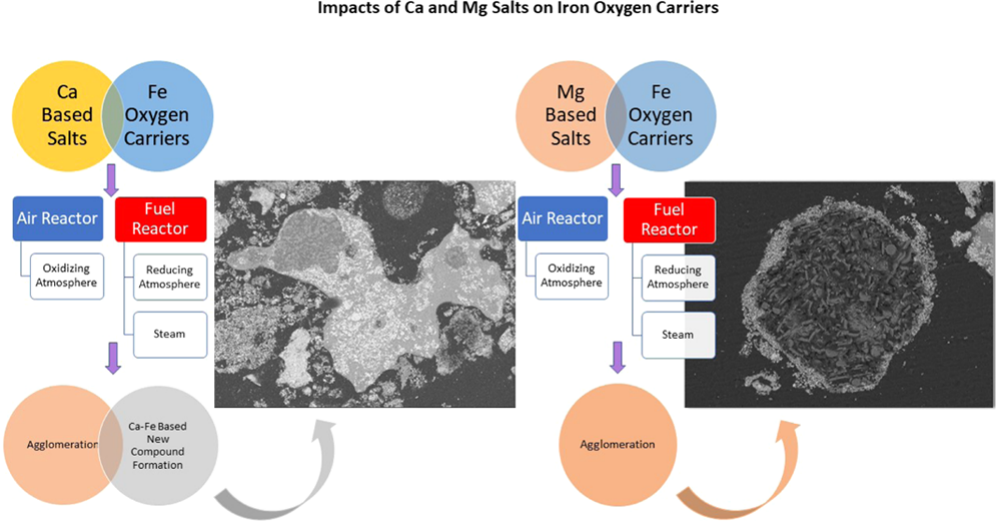

Chemical looping combustion (CLC) is one of the most promising methods for carbon capture and storage (CCS). An oxygen carrier, i.e., a mineral that can be oxidized and reduced, is used to convert the fuel in the process. The produced CO_2_ is inherently separated from the air components that enables easier CCS. The use of biomass-based fuels is desirable since it can lead to negative CO_2_ emissions. On the other hand, alkali compounds from the biomass may interact with the oxygen carrier causing problems, such as deactivation of the oxygen carrier. The most common oxygen carriers contain iron, since iron-based ores and industrial waste materials are readily available and cost-efficient. Therefore, the interaction between the iron oxygen carriers and the biomass ash-forming compounds needs to be investigated. Since Ca/Mg are abundant in biomass, it is important to clarify how their compounds interact with the oxygen carrier. In this study, the effect of Ca/Mg carbonates, chlorides, nitrates, sulfates, and phosphates along with synthetic biomass-derived ash on iron oxides was investigated. Redox reactions were investigated at 950 °C during 5 h under both oxidizing and reducing atmospheres. The results showed that the effect of Ca/Mg salts on the oxygen carrier varied depending on the anion of the salt. Generally, the nitrate- and phosphate-based salts of both Ca and Mg showed the harshest effect regarding agglomeration of the oxygen carriers. It was shown that the Ca/Mg-based compounds interacted differently with iron oxides, which was an unexpected result.

## 1 Introduction

Chemical looping combustion (CLC) is one of the most promising methods to reduce the cost of CO_2_ capture to tackle the climate change.^1^ CLC enables us to separate CO_2_ from other combustion products; therefore, there is no energy consumed for the gas separation.^2^ A CLC process consists of two interconnected fluidized beds: a fuel reactor and an air reactor.^3^ In the fuel reactor, the fuel is oxidized by a solid oxygen carrier, which gets reduced. In the air reactor, the oxygen carrier is oxidized by air. Generally, the oxygen carriers are chosen from oxides of Cu, Fe, Mn, and their oxide-based combinations or ores.^4−7^ Even though gaseous fuels are most commonly used in CLC systems, solid fuels can be favorable since they are less expensive and more abundant.^8,9^ Recently, the use of biomass in CLC has attracted great attention since it gives a possibility to achieve “negative CO_2_ emission” goals.^10,11^ There is no doubt that the use of biomass in CLC systems brings a lot of advantages.^12,13^ However, biomass-derived ash consists of highly reactive species such as alkali metal compounds and compounds of alkaline earth elements,^13−15^ which may cause partial sintering or agglomeration of the oxygen carriers.^16^

If the interaction mechanism between ash-forming matters and the oxygen carrier can be understood, selection of the most appropriate biomass source as a fuel for CLC applications can be made. Ash-forming species may exist as different compounds, and their composition in a biomass may vary depending on the source of biomass.^14,15^ In the literature, studies focused on the interaction of alkali-metal-based ash-forming matters and oxygen carriers have been published.^17−19^ Especially, the effect of alkali metal salts/alkaline earth salts has been focused on by researchers, as the salts can affect the oxygen carriers in various ways.^16,20−24^ Among these salts, the interaction of Ca- and Mg-based ones with the oxygen carrier has not been investigated in detail, since their effect on the oxygen carriers was expected to be similar.^15^ A recent study showed, however, that their impact might differ depending on which oxygen carrier was used.^25^ The Ca compounds showed higher reactivity toward iron oxides than the Mg compounds did, and the formation of Ca–Fe-based oxides was observed. On the contrary, no formation of Mg–Fe oxides occurred.^25^ It is known that the Ca/Mg ratio in biomass can vary from one biomass to another (Table 1). From this point of view, it is important to clarify more the effects of Ca- and Mg-based species on iron oxygen carriers, since iron oxides are one of the most commonly used oxygen carriers in combustion of biomass.^26^ In this way, the right fuel selection can be made among the several biomass sources available. In addition to this, it is important to reveal the interaction mechanisms of the alkaline earth compounds with the other ash-forming matters. Combinations of alkali metal compounds and silica, for example, can cause serious agglomeration since alkali metal silicates may have melting points that are lower than the operation temperature of the combustor.^13,16,27−30^ In addition to the risk of agglomeration due to ash melts, there is a risk of deactivation of oxygen carriers via the formation of new compounds during CLC operation. Moreover, thermodynamic and kinetic characteristics of the redox reactions may be different depending on which oxygen carrier is used.^31,32^

**Table 1:** Ratio of Ca to Mg in Different Biomass Sources (Oxide-Based Weight Ratio)^33^

	seeds/hulls	straw grasses	agricultural (other)	algae	bark	cardoon	forest residue	torrefied wood	wood
Ca/Mg	2.6	4.3	2.3	0.4–1.8	8.3	5.3	6.0	3.7	5.5

So far, there is no study published that presents data from investigations of the high-temperature interactions between alkaline earth element compounds common in biomass ash-forming matter and pure Fe oxygen carriers, individually. Therefore, it was considered important to investigate these interactions to make it possible to select the best oxygen carrier and type of biomass to be used in real applications. In this way, problematic sintering and formation of unwanted compounds can be avoided.

In the present study, the interactions between pure Fe oxides and Ca/Mg-based compounds (representing reactive biomass ash species) were investigated. Two synthetic model ashes consisting of either Ca- or Mg-based compounds were also prepared and used in experiments with Fe oxides to investigate the overall effect of the alkali earth metal species on the Fe oxides. Thermodynamic equilibrium calculations were used for theoretical evaluation of the chemical systems and comparison with the compounds obtained in the experiments. Agglomeration and sintering of the oxygen carriers caused by the alkali earth compounds was studied by visual observation and determination of the surface area of the samples after the experiments.

## 2 Results and Discussion

### 2.1 Effect of Ca/Mg Hydroxides on the Pure Fe Oxygen Carriers

**Figure 1:**
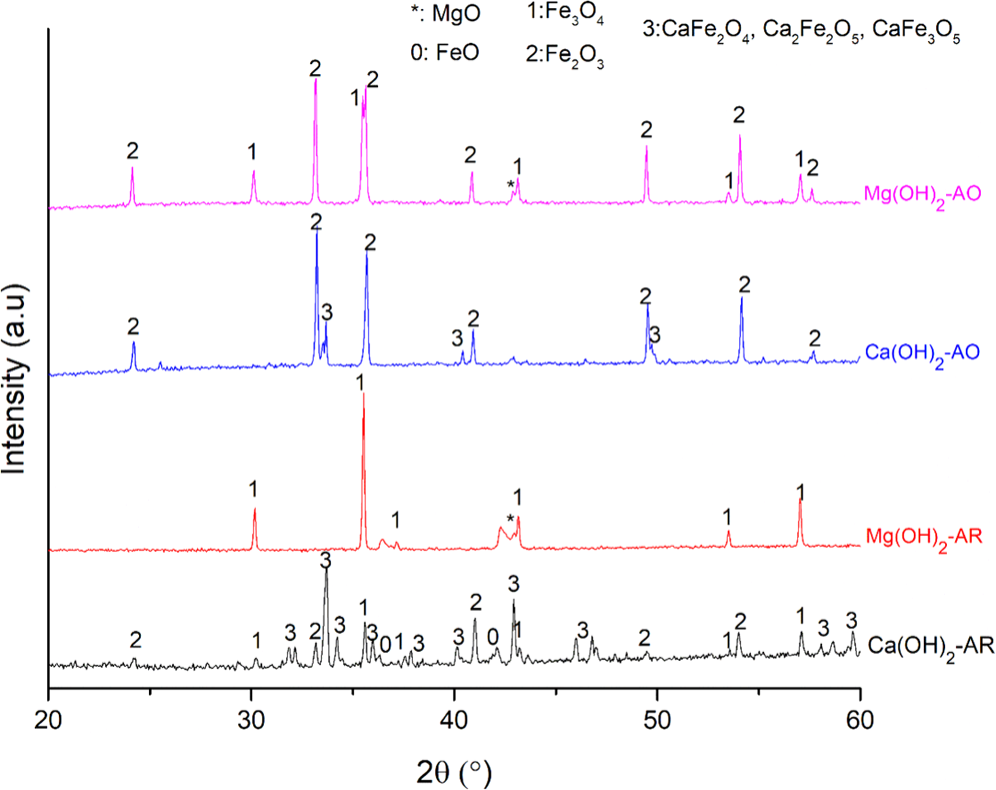
XRD patterns of the iron oxides—Ca(OH)_2_ and Mg(OH)_2_ mixtures after the experiments (AR: after reduction, AO: after oxidation).

Table 2 shows the results of the experiments (Figure 1) and thermodynamic equilibrium calculations. When Ca(OH)_2_ was used as an alkaline earth compound representative in the mixture, the formation of new compounds, such as CaFe_2_O_4_ and Ca_2_Fe_2_O_5_, was observed under both reduction and oxidation conditions most likely via reactions 1 and 2. These reactions are only solid-state reactions and do not involve reduction of the iron. As both new compounds formed have been reported as potential oxygen carriers for energy-related applications, their formation as such was not considered as a problem.^34,35^ However, a serious agglomeration was observed, particularly during the oxidation step, most likely due to solid-state formation of these compounds. It is known that CaFe_2_O_4_ is stable under oxidizing atmosphere, and it may dissociate into Ca_2_Fe_2_O_5_ and Fe_3_O_4_ under reducing atmosphere (reaction 3). This was also observed in the X-ray diffraction (XRD) analysis of the reduced Ca(OH)_2_–Fe_2_O_3_ mixture.
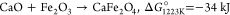
1
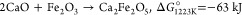
2
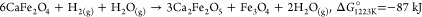
3

**Table 2:** Products of Interaction between Iron-Based Oxygen Carriers and Calcium and Magnesium Hydroxides

	experimental results			thermodynamic calculation results	
alkaline earth compounds	oxidation Fe_3_O_4_ as starting material	reduction Fe_2_O_3_ as starting material	visual inspection	reduction Fe_3_O_4_ as starting material	reduction Fe_2_O_3_ as starting material
Ca(OH)_2_	Fe_2_O_3_	Fe_3_O_4_	small agglomerates	Fe_2_O_3_	Fe_3_O_4_
	CaFe_2_O_4_	Fe_2_O_3_		CaFe_2_O_4_	Ca_2_Fe_2_O_5_
		Ca_2_Fe_2_O_5_			
		FeO			
		CaFe_3_O_5_			
Mg(OH)_2_	Fe_2_O_3_	Fe_3_O_4_	low-grade fine agglomerates	Fe_2_O_3_	Fe_3_O_4_
	Fe_3_O_4_	FeO		MgO	MgO
	MgO	MgO		Mg*_x_*Fe_2–*x*_O_4_^a^	Mg_x_Fe_3–*x*_O_4_^a^

a Trace amount.

Similarly, the presence of Mg(OH)_2_ also caused an agglomeration of the sample. However, the observed agglomeration was milder in this case. There was no significant Mg- and Fe-based oxide formation observed based on the XRD analysis of the reaction product. This is not surprising since reaction 4 is thermodynamically favorable only below 700 °C. Above 700 °C, MgFe_2_O_4_ may dissociate into MgO and Fe_2_O_3_ under oxidizing atmosphere.
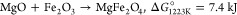
4

### 2.2 Effect of Ca-Based Salts on the Pure Fe Oxygen Carriers

**Figure 2:**
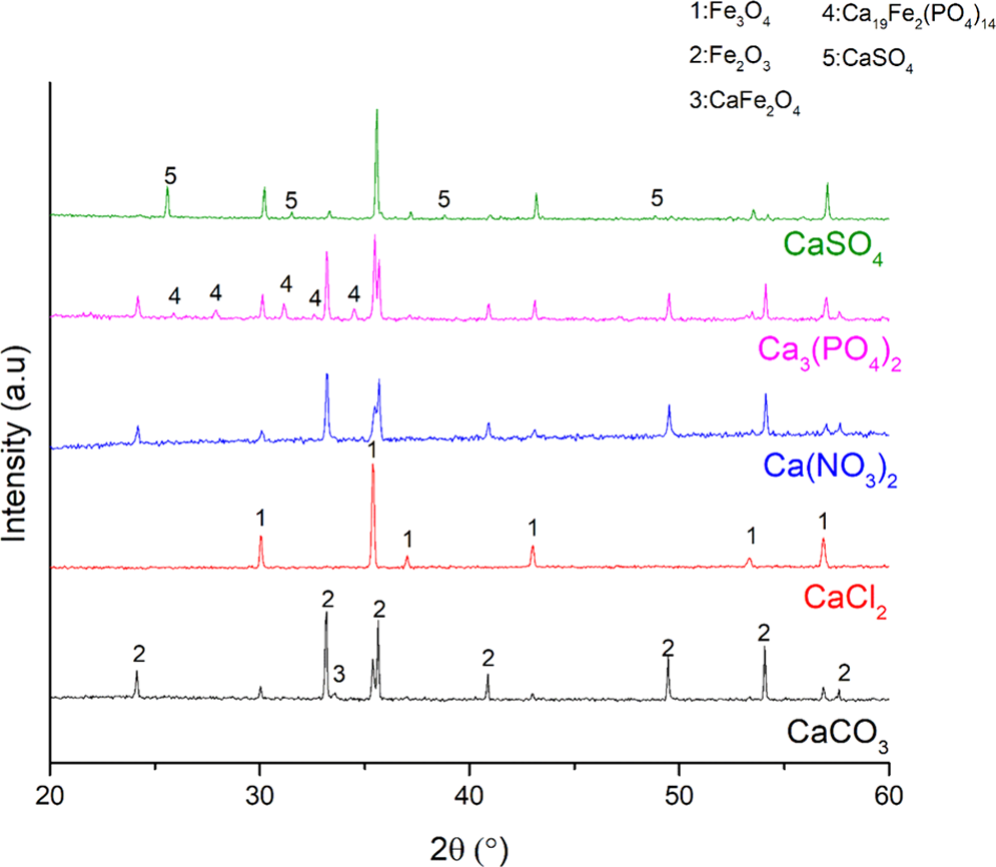
XRD patterns of the mixtures of iron oxygen carriers and Ca salts after the reduction. The chemical formula of the calcium salt is used for the identification of the respective product diffraction data.

**Figure 3:**
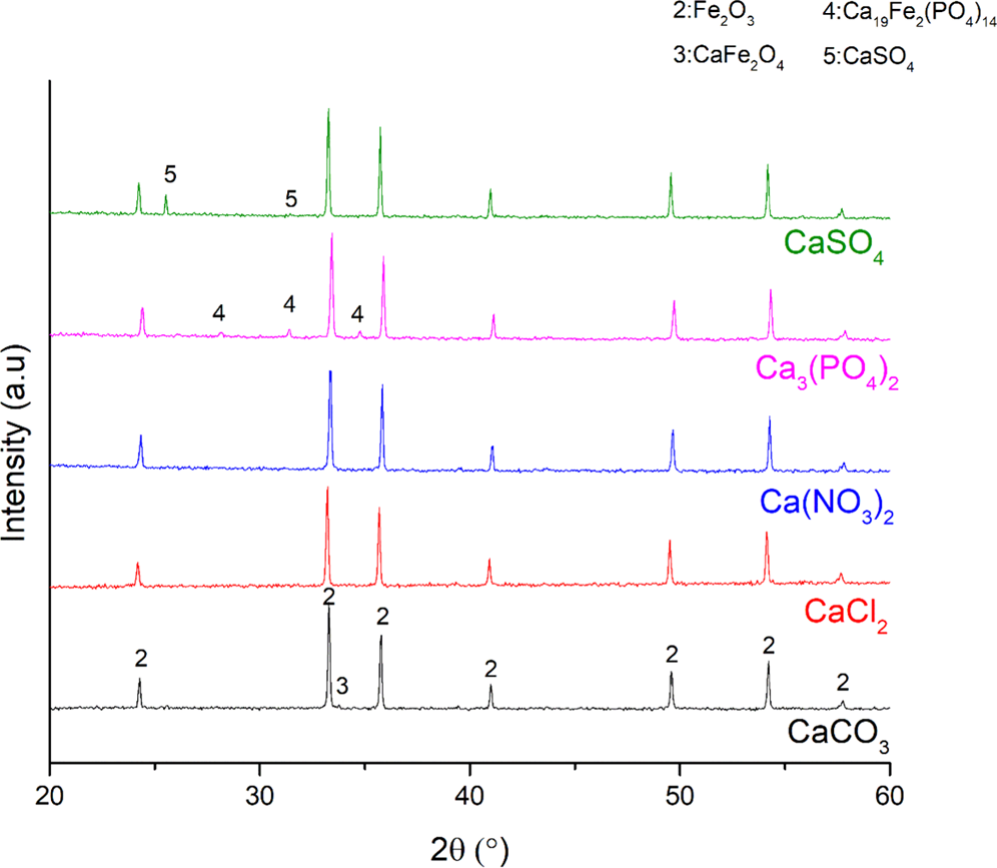
XRD patterns of the mixtures of iron oxygen carriers and Ca salts after the oxidation. The chemical formula of the calcium salt is used for the identification of the respective product diffraction data.

Some types of biomass sources such as bark and forest residue can contain a high amount of Ca-based species.^15^ Since alkaline earth metal compounds can easily interact with the other ash-forming matters present in biomass, their effect on the oxygen carriers has attracted the attention of researchers.^21,36−38^ Therefore, it is important to reveal the interaction between the alkali-earth-based salts and Fe oxygen carriers. The alkaline earth elements are mainly present in the biomass ashes as chlorides, carbonates, sulfates, and phosphates.^39^ Especially calcium carbonates were found as deposits in biomass-fired power plants.^40^ Thermodynamic equilibrium calculations showed that CaFe_2_O_4_ and Ca_2_Fe_2_O_5_ were expected along with iron oxides in redox reactions of calcium carbonate–iron oxide mixtures (Table 3). XRD analysis results (Figures 2 and 3 for reduction and oxidation, respectively) showed the presence of CaFe_2_O_4_ both after the reduction and oxidation experiments with the CaCO_3_–Fe oxide mixtures. This result was expected since CaCO_3_ is stable up to 750 °C^41^ and can interact with Fe oxides to form CaFe_2_O_4_ through reaction 5. Most likely, reactions 6 and 1 occurred above 750 °C to form CaFe_2_O_4_. Along with the CaFe_2_O_4_ formation, a strong agglomeration was observed when CaCO_3_ interacted with the Fe oxides both under oxidizing and reducing atmospheres. This agglomeration most likely occurred due to the CaFe_2_O_4_ formation by solid-state reactions. It is known that the dwell during the redox reactions can cause sintering via grain growth.^42^ In addition to that, calcium ferrites were detected in the sintered matrix of the iron ores in different studies.^43,44^
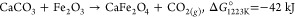
5
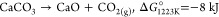
6

**Table 3:** Compounds Identified as Products of Interaction between Fe Oxygen Carriers and Ca Salts

	experimental results			thermodynamic calculations	
calcium salts	oxidation Fe_3_O_4_ as starting material	reduction Fe_2_O_3_ as starting material	visual inspection	oxidation Fe_3_O_4_ as starting material	reduction Fe_2_O_3_ as starting material
CaCO_3_	Fe_2_O_3_	Fe_3_O_4_	high-grade coarse agglomerates	Fe_2_O_3_	Fe_3_O_4_
	CaFe_2_O_4_	Fe_2_O_3_		CaFe_2_O_4_	Ca_2_Fe_2_O_5_
		CaFe_2_O_4_			
CaCl_2_	Fe_2_O_3_	Fe_3_O_4_	high-grade agglomerates	Fe_2_O_3_	Fe_3_O_4_
				CaFe_2_O_4_	Ca_2_Fe_2_O_5_
				CaCl_2_ (liq.)	
Ca(NO_3_)_2_	Fe_2_O_3_	Fe_3_O_4_	high-grade coarse agglomerates	Fe_2_O_3_	Fe_3_O_4_
		Fe_2_O_3_		CaFe_2_O_4_	Ca_2_Fe_2_O_5_
		amorphous phase			
Ca_3_(PO_4_)_2_	Fe_2_O_3_	Fe_3_O_4_	high-grade coarse agglomerates	Fe_2_O_3_	Fe_3_O_4_
	Ca_19_Fe_2_(PO_4_)_14_	Fe_2_O_3_		Ca_3_P_2_O_8_	Ca_5_(PO_4_)_3_(OH)
		Ca_19_Fe_2_(PO_4_)_14_			Fe_3_P_2_O_8_
CaSO_4_	Fe_2_O_3_	Fe_3_O_4_	mild agglomerates	Fe_2_O_3_	Fe_3_O_4_
	CaSO_4_	CaSO_4_		CaSO_4_	Ca_2_Fe_2_O_5_

When the impact of chloride ions on oxygen carrier was investigated earlier by researchers, the results showed that HCl formation may cause a deactivation of the oxygen carriers due to the formation of gaseous products from reaction between oxygen carrier and Cl-based species.^45^ However, high steam concentration in the CLC process was successful to prevent this reaction and formation of HCl was observed instead. In the investigation of the effect of CaCl_2_ as a Cl source on the iron oxygen carriers in the present work, the theoretical calculations indicated the formation of CaFe_2_O_4_/Ca_2_Fe_2_O_5_, but no such products were observed in the experiments. This was most likely due to the fact that the melting point of CaCl_2_ (772 °C) is lower than the operation temperature and melted CaCl_2_ probably interacted with the alumina crucible to form a Ca–Al oxide on the crucible surface via reaction 7. The melting temperatures of the chloride–hydrates are even lower than that of the chloride. Due to the melt formation, agglomeration of the oxygen carriers was also observed.
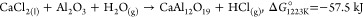
7

When the nitrate was used as a calcium source, the formation of CaFe_2_O_4_/Ca_2_Fe_2_O_5_ was not observed as in the CaCl_2_ case experiments. The melting temperature of Ca(NO_3_)_2_ is around 560 °C and its hydrate’s melting temperatures are even lower. From this point of view, most likely the same scenario as in the CaCl_2_ case occurred in the Ca(NO_3_)_2_ experiment and a low-viscosity melt flowed through the sample powder bed and reacted with the alumina crucible. Similar to the CaCl_2_ case, a strong agglomeration of the iron oxide occurred in the experiment with the nitrate. This agglomeration was the strongest compared to those caused by the other Ca-based salts. During the reduction experiment, an amorphous phase was also observed, which may be related to a melt formation on the crucible.

When Ca_3_(PO_4_)_2_ was used in the mixture, the formation of Ca_19_Fe_2_(PO_4_)_14_ was observed under both oxidizing and reducing atmospheres, despite the results obtained in the thermodynamic calculations, which means that this compound may be stable under high temperatures and different atmospheres. The formation of this product was accompanied by some agglomeration of the mixture as well. In the literature, Ca_19_Fe_2_(PO_4_)_14_ is reported as a glaze component, so there is a risk that it can lead to a deactivation of oxygen carriers via the formation of a glassy phase.^46^

CaSO_4_ is a comparably stable salt under operation conditions, and it has a higher melting point than other Ca-based salts used in the study. CaSO_4_ can be reduced to CaS in the presence of reducing atmospheres such as H_2_ and CO. However, reductive potential (PH_2_/pH_2_O) is known to be one of the most important parameters to decide the decomposition mechanism of CaSO_4_.^47^ Since steam was also present under reducing atmosphere in this study, although the amount was very small, CaSO_4_ could be observed in the system after the reduction. No new Ca–Fe-based oxide or other reaction product was observed in the experiments, which is also consistent with the results provided by Thermodynamic Equilibrium Calculations (TEC).

To summarize, the most dramatic effects regarding the agglomeration formation were observed in the mixtures of iron oxygen carriers and Ca carbonate, nitrate, and phosphates. If the formation of Fe-based new compounds is taken into consideration, calcium phosphate generated the harshest conditions for the Fe oxygen carrier. From this point of view, the best biomass for Fe-based oxygen carriers can be chosen from the biomasses with the highest CaO/P_2_O_5_ value, such as wood-derived biomass,^33^ to limit the formation of Ca–Fe–P-based oxides. Calcium phosphates can also interact with the other compounds present in the biomass, especially with alkali metal compounds.^48^ However, it is worth noting that the melting temperatures of calcium phosphates may be close to the operation temperature, which shows that there is a risk for agglomeration of the bed material.^49^

### 2.3 Effect of Mg-Based Salts on Pure Fe Oxygen Carriers

**Figure 4:**
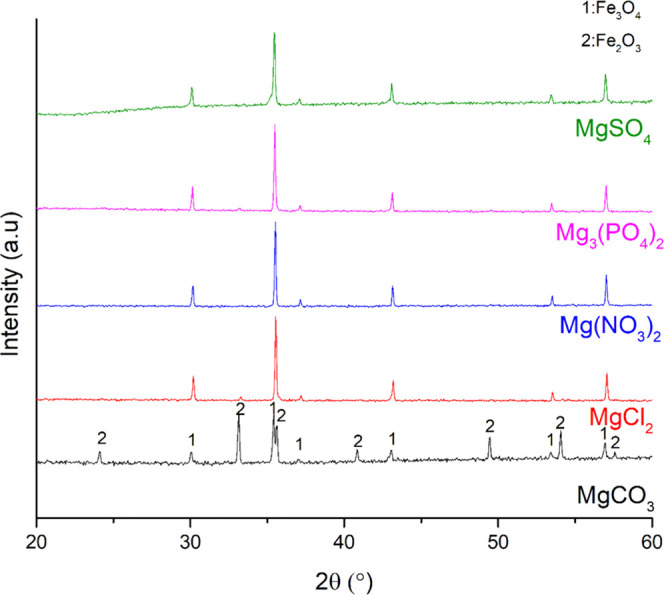
XRD patterns of the mixtures of iron oxygen carriers and Mg salts after reduction.

**Figure 5:**
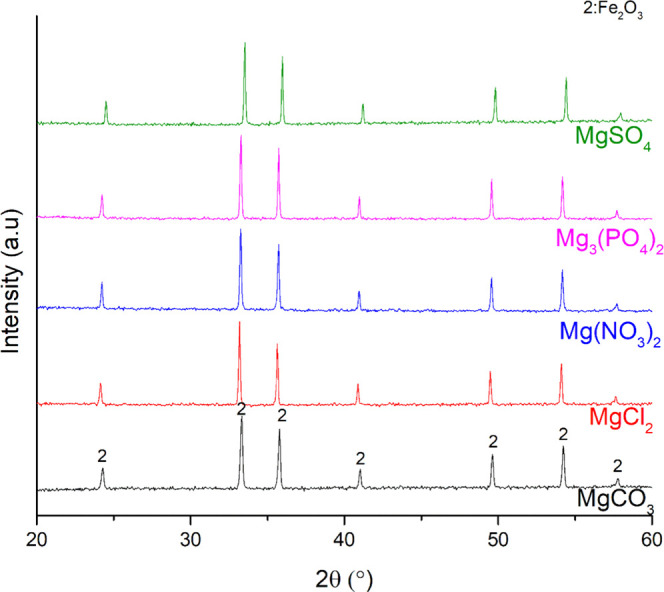
XRD patterns of the mixtures of iron oxygen carriers and Mg salts after oxidation.

The concentrations of Mg compounds are generally lower than those of the Ca compounds in biomass.^50^ However, there are some biomass sources such as chlorella, spirulina, coffee residue, and bean curd, which contain more Mg than Ca.^51^ Therefore, it is important to have a better understanding on the interaction of Mg-based salts and the oxygen carriers. It is known that the decomposition temperature of alkaline earth carbonates to oxides decreases down the group in the periodic table.^52^ When MgCO_3_ was used as a salt compound in the Fe oxide–salt mixture, no formation of new Fe compounds was observed in any of the experiments (Figures 4 and 5). The decomposition of MgCO_3_ to MgO is thermodynamically favorable above 400 °C (reaction 8). Therefore, MgCO_3_ will exist in the mixture as MgO above 400 °C, and it may interact with Al_2_O_3_ rather than Fe oxide as indicated by thermodynamics (reactions 9 and 10). The system was assumed as a closed system in TEC, and no interaction with the Al_2_O_3_ crucible was considered. Therefore, the modeling results differed from the experimental ones (Table 4).
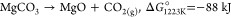
8
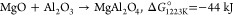
9
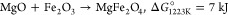
10

**Table 4:** Obtained Results of Interaction between Fe Oxygen Carriers and Mg-Based Compounds

	experimental results			thermodynamic calculations	
magnesium salts	oxidation Fe_3_O_4_ as starting material	reduction Fe_2_O_3_ as starting material	visual inspection	oxidation Fe_3_O_4_ as starting material	reduction Fe_2_O_3_ as starting material
MgCO_3_	Fe_2_O_3_	Fe_3_O_4_	high-grade agglomerates	Fe_2_O_3_	Fe_3_O_4_
		Fe_2_O_3_		MgO	Mg*_x_*Fe_2–*x*_O_4_, *x* < 2
		amorphous phase			
MgCl_2_	Fe_2_O_3_	Fe_3_O_4_	low-grade agglomerates	Fe_2_O_3_	Fe_3_O_4_
		Fe_2_O_3_		MgO	Mg*_x_*Fe_2–*x*_O_4_, *x* < 2
Mg(NO_3_)_2_	Fe_2_O_3_	Fe_3_O_4_	high-grade coarse agglomerates	Fe_2_O_3_	Fe_3_O_4_
				MgO	Mg*_x_*Fe_2–*x*_O_4_, *x* < 2
Mg_3_(PO_4_)_2_	Fe_2_O_3_	Fe_3_O_4_	high-grade coarse agglomerates	Fe_2_O_3_	Fe_3_O_4_
		Fe_2_O_3_		Mg_3_P_2_O_8_	Mg_3_P_2_O_8_
					Fe_3_P_2_O_8_
MgSO_4_	Fe_2_O_3_	Fe_3_O_4_	high-grade agglomerates	Fe_2_O_3_	Fe_3_O_4_
		amorphous phase		MgO	Mg*_x_*Fe_2–*x*_O_4_, *x* < 2

In the case of MgCl_2_, neither serious agglomeration nor formation of any new Fe compound was observed. This result is not unexpected since reaction 11 is the most thermodynamically favorable at temperatures above 540 °C.
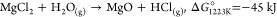
11

Mg(NO_3_)_2_ and Mg_3_(PO_4_)_2_ affected the Fe-based oxygen carriers harsher. A serious agglomeration was observed after both reduction and oxidation experiments, although there was no new Fe compound observed. This result was not expected for Mg_3_(PO_4_)_2_ in terms of agglomeration as it has a much higher melting point than the other salts used in this study (1184 °C).^53^ Mg(NO_3_)_2_ has a lower melting point than the operation temperature,^54^ and an agglomeration also occurred in the nitrate–Fe oxide system.

When MgSO_4_ was used as a salt compound in the experiments, a milder agglomeration was observed compared to the cases with the nitrate and phosphate-based salts. There was no Fe-based new crystalline compound formed; however, a visible amount of amorphous phase was detected in the phase analysis after the reduction experiment.

### 2.4 Effect of Ca-/Mg-Salt-Based Synthetic Ash on the Pure Fe Oxygen Carriers

**Figure 6:**
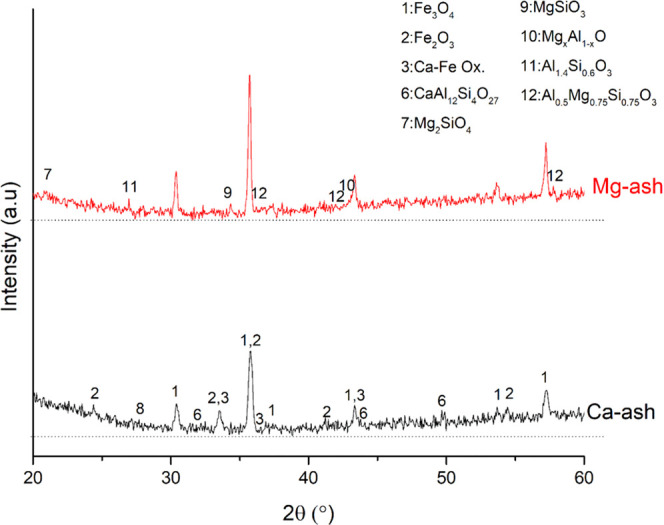
XRD patterns of the mixtures of iron oxygen carriers and Ca/Mg salts containing synthetic ashes after reduction.

**Figure 7:**
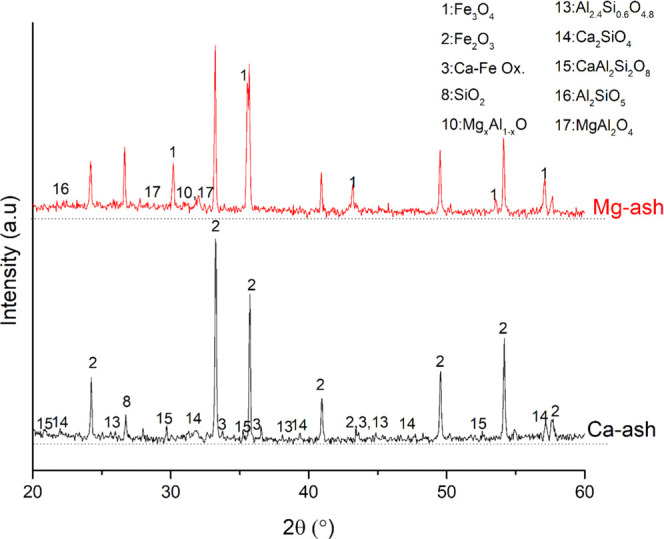
XRD patterns of the mixtures of iron oxygen carriers and Ca/Mg salts containing synthetic ashes after oxidation.

Redox reactions between Fe oxides and synthetic ash mixtures, which consisted of either Ca- or Mg-based salts as described in the Materials and Methods section, were studied. The detailed chemical composition that was used to prepare the synthetic ash can be found in Section 4. The experimental results can be found in Figure 6 for the reduction experiments, and in Figure 7 for the oxidation experiments. In addition to this, thermodynamic calculation results are given in Table 5 for comparison between the experimental results and theoretical equilibrium compositions. The most significant result was the formation of Ca–Fe-based oxides in the cases where only Ca-based salts (combination of Ca carbonate, chloride, nitrate, phosphate, and sulfate) were used as a salt component of the synthetic ash. The formation of Ca–Fe-based oxides was observed both under reducing and oxidizing atmospheres, even though there was no such formation indicated by TEC results. Along with Ca–Fe oxides, calcium silicate and calcium aluminum silicate formation were also observed, which is very common to form during the combustion of biomass fuels.^55,56^ When the combination of Mg-based salts was used in the synthetic ash, there was no formation of Mg–Fe oxides observed. This result is important since it shows that there was no direct chemical interaction between the Mg-based salts and the Fe oxygen carrier. However, a harsher agglomeration occurred than in the case of Ca compounds, most likely due to the lower melting temperatures of the Mg salts than those of the Ca salts. Except the formation of Ca–Fe oxides, there was no problematic new solid-phase formation observed, which may cause deactivation of the oxygen carriers.

**Table 5:** Obtained Results of Interaction between Fe Oxygen Carriers and the Synthetic Ash Containing either Ca- or Mg-Based Salts as Alkali Earth Content

	experimental results			thermodynamic calculations	
used salt combination	oxidation	reduction	visual inspection	oxidation	reduction
Ca-based	Fe_2_O_3_	Fe_3_O_4_	severe agglomerates	Fe_2_O_3_	Fe_3_O_4_
	Ca–Fe oxides	Fe_2_O_3_		Ca_3_Si_2_O_7_	Ca_2_SiO_4_
	Ca_2_SiO_4_	Ca–Fe oxides		CaFe_2_O_4_	Na_3_PO_4_
	SiO_2_	Ca–Al–Si oxides		K(Na)AlSiO_4_	Na_2_CaSiO_4_
	Ca–Al–Si oxides	SiO_2_		K_2_SO_4_	K(Na)AlSiO_4_
	Al–Si oxides	amorphous phases		KCl (liq.)	
				Na_3_PO_4_	
Mg-based	Fe_2_O_3_	Fe_3_O_4_	severe agglomerates	Fe_2_O_3_	Fe_3_O_4_
	Fe_3_O_4_	MgSiO_3_		Mg_2_SiO_4_	Mg_2_SiO_4_
	Al_2_SiO_8_	Mg_2_SiO_4_		NaAlSiO_4_	NaAlSiO_4_
	MgAl_2_O_4_	Mg–Al–Si oxides		KCl (liq.)	Na_3_PO_4_
	SiO_2_	Al–Si oxides		Na_3_PO_4_	K_2_SO_4_
	amorphous phases	Mg_1–*x*_Al*_x_*O, *x* < 0.5		K_2_SO_4_	
		SiO_2_			
		amorphous phases			

The thermodynamic calculation results differed from the experimental results for both reducing and oxidizing atmospheres. This may be expected since the volatile species are assumed to stay in the system in the theoretical modeling of the systems. However, the flowing gas in the experimental system (air for the oxidation experiments and H_2_/Ar/H_2_O mixture for the reduction experiments) can sweep away the volatile species (mainly easily volatilized salts in this work) and change the chemistry. In addition to this, XRD has a detection limit around ca. 1–2% by volume, and this limit depends on the density, atomic number of the elements in the sample, and the crystal structure of the present phases. If the amount of the phase is lower than this limit, the peaks belonging to the phase will not be detected in the diffraction pattern or will remain under the background.^57^

**Figure 8:**
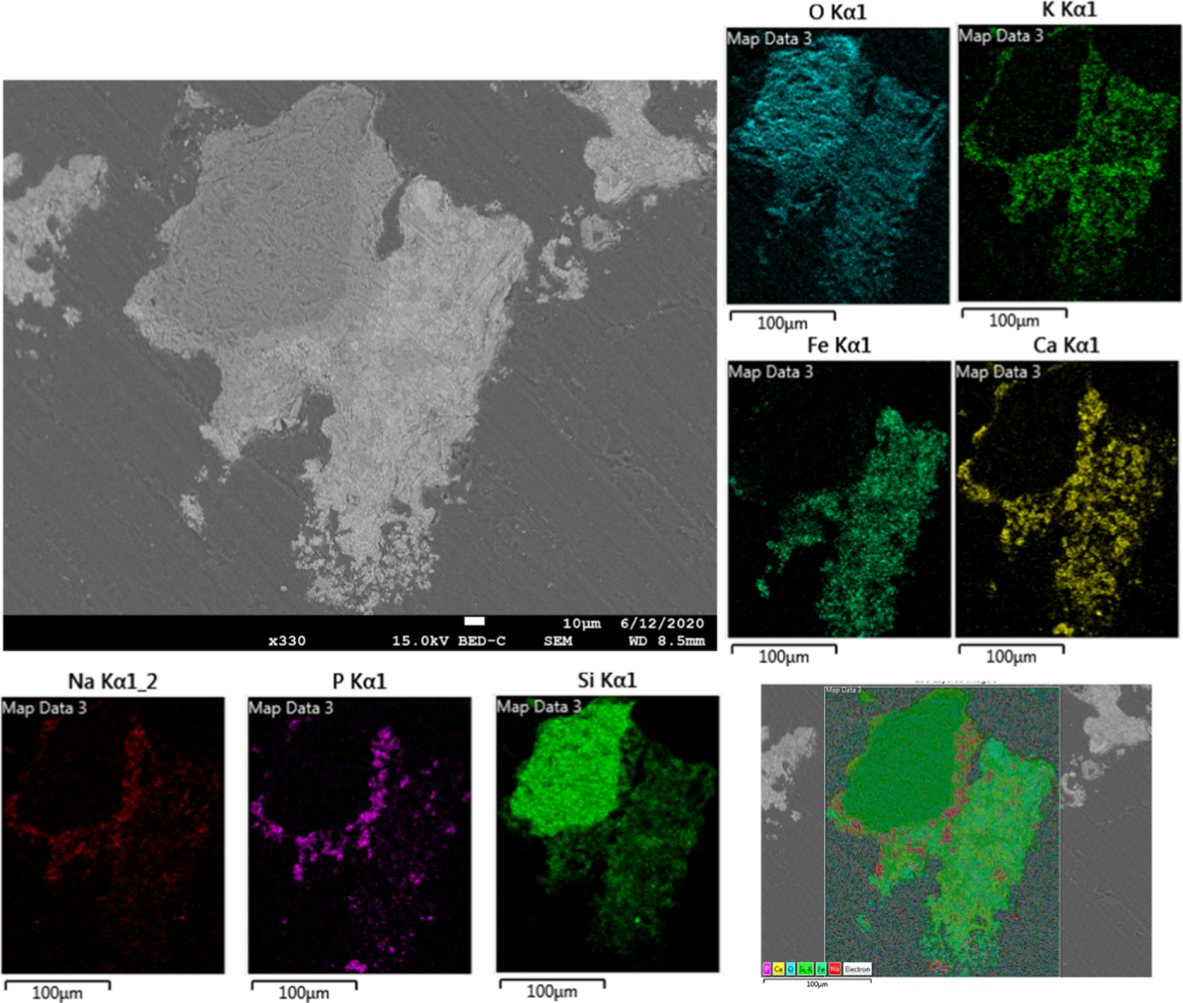
SEM micrograph (BSE) and elemental mapping of the cross section of the sample consisting of Ca-salt-based synthetic ash and iron oxide after the experiment.

**Figure 9:**
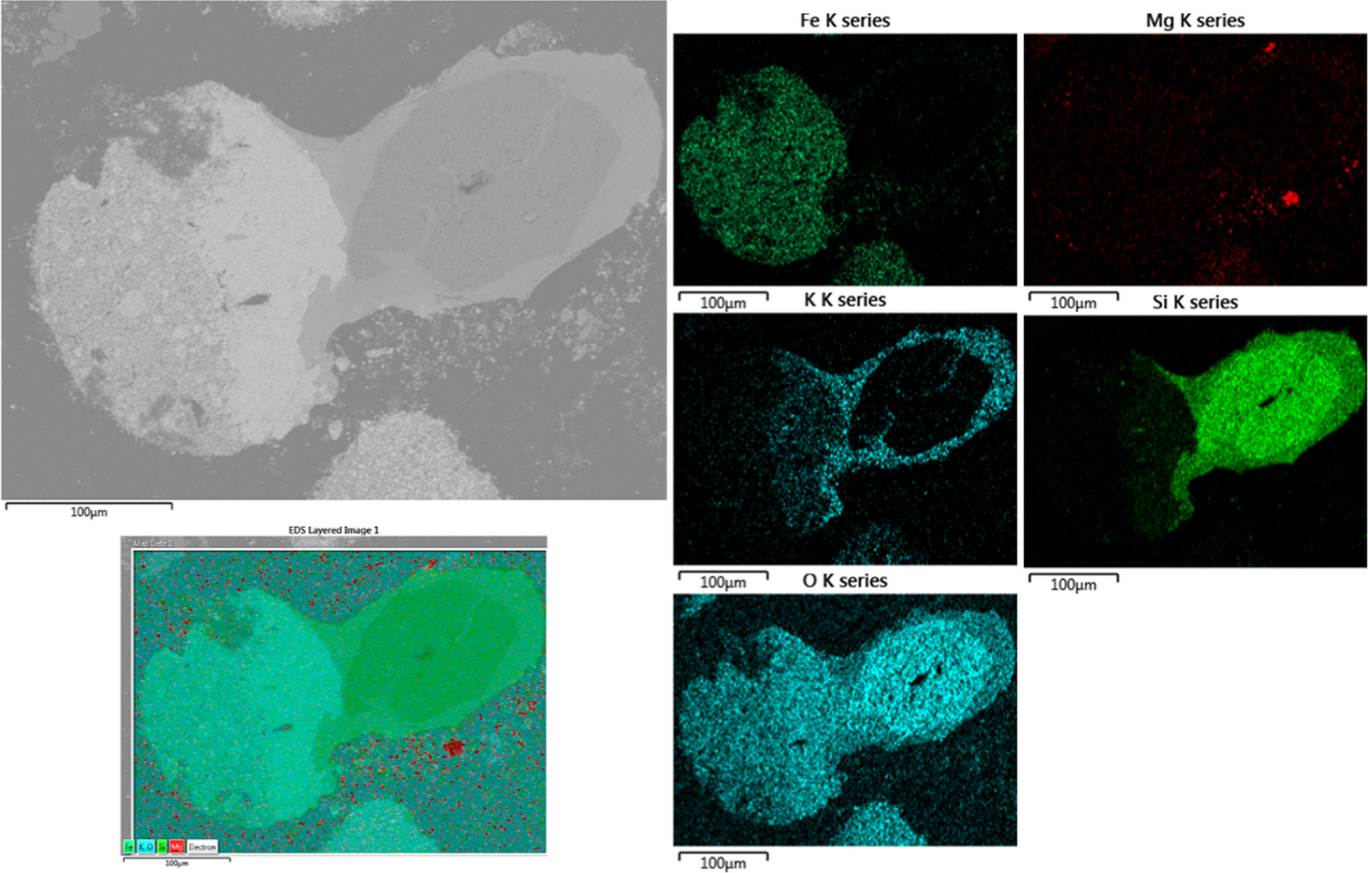
SEM micrograph (BSE) and elemental mapping of the cross section of the sample consisting of Mg-salt-based synthetic ash and iron oxide after the experiment.

Another important information that these experiments can give is where in or on the oxygen carrier particles the Ca- and Mg-based species are located. The formation of bridges consisting of more or less melted silicates between oxygen carrier particles causes serious agglomeration problems in the CLC process.^25^ Therefore, the elements and compounds that may contribute to such bridge formation in the reaction systems studied here were investigated more in detail. To reveal the independent effects of Ca- or Mg-based salts in the synthetic ash, elemental mapping by scanning electron microscopy–energy-dispersive X-ray spectroscopy (SEM–EDX) was made of the samples after the redox experiments. Figure 8 shows the elemental mapping and SEM micrograph of the cross section of the sample consisting of Ca-salt-based synthetic ash and iron oxide after the experiment. An agglomerated oxygen carrier particle was chosen here. It was found that a silicon dioxide particle was stuck to the iron oxide via a bridge phase of the type which was mentioned before. The silicon oxide was surrounded by a thin layer containing K and Ca. This layer most likely belongs to a potassium silicate–potassium calcium silicate mixture phase, which is the common phase where the ash contains a high amount of Ca.^58^ Moreover, the outer layer of the bridge phase, which was closer to the iron oxides, consisted of Na, P, and Ca, which indicates the formation of an alkali phosphate. The most significant effect of Ca-based salts in this case was the formation of Ca–Fe oxides, which could easily be observed in the elemental mapping. When the same analysis was carried out for Mg-based ash, the results were significantly different (Figure 9). A silicon-rich particle was stuck to the iron oxide like in the Ca case; however, there was no relation between the outer phase layer of the silicon-based particle and Mg. As expected, potassium silicate was the bridge phase, which caused the agglomeration of the oxygen carrier. Moreover, there was no correlation between Mg and Fe oxides, which was different from the Ca case. To verify this distinctive interaction mechanism between Fe oxides and Ca- or Mg-based salts, one more experiment was carried out. In the experiment, Fe oxygen carriers were mixed with a synthetic ash consisting of both Ca- and Mg-based salts.

**Figure 10:**
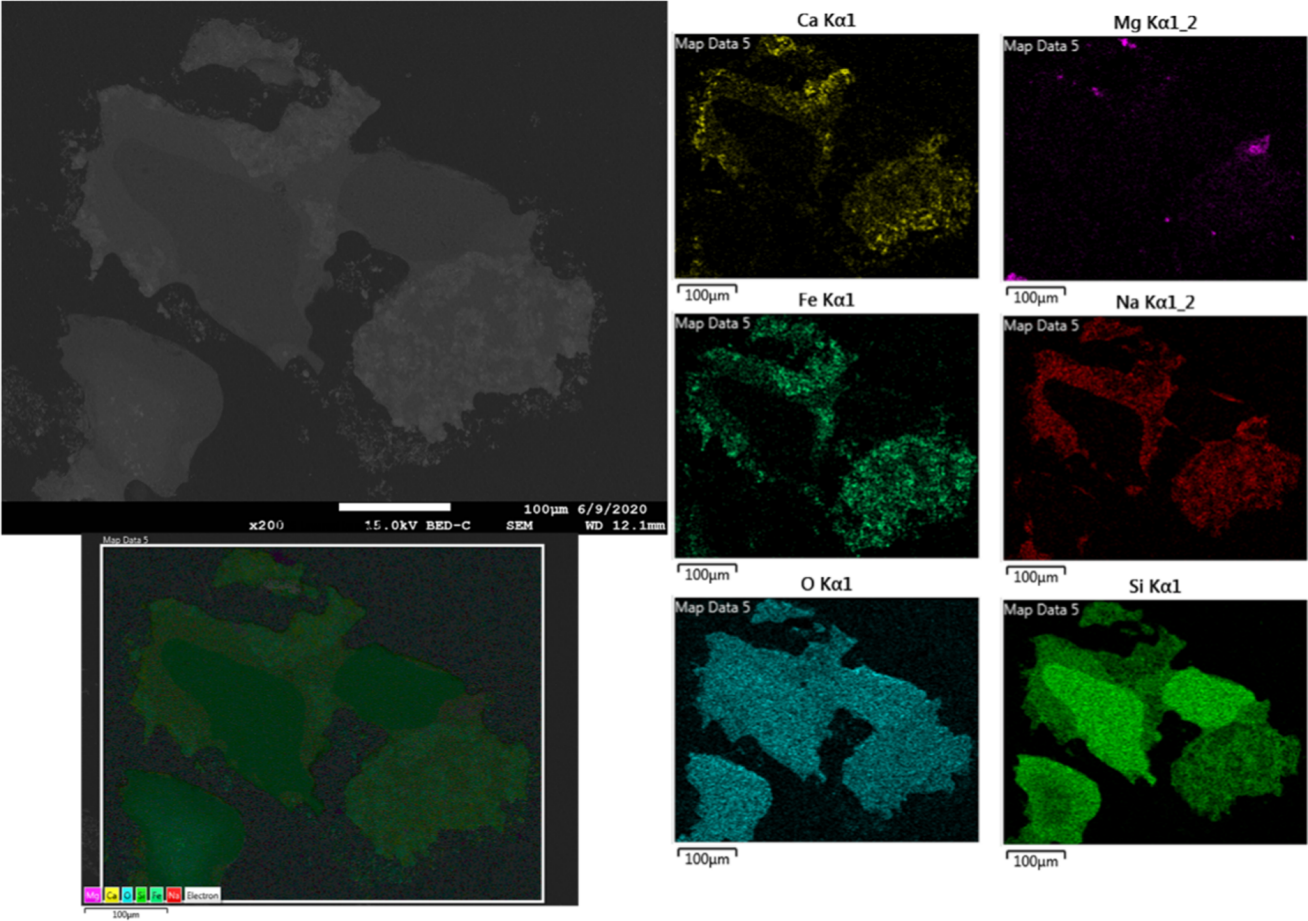
SEM micrograph (BSE) and elemental mapping of the cross section of the sample consisting of both Ca- and Mg-salt-based synthetic ash and iron oxide after the experiment.

A significant agglomeration was observed in the mixture, which was expected due to the use of both Ca- and Mg-based salts along with alkali metal compounds (Figure 10). Silicon oxide particles were observed within the darker parts of the cross section and found to be attached to the iron oxides. Fe oxides were surrounded by Ca- and Na-based oxides along with Si. However, there was no significant correlation observed for the interaction of Mg and Fe oxides. Likewise, there was no similar interaction observed between Mg or Ca with other ash-forming matters, indicating selective phase formation. This result was quite surprising since Ca- and Mg-based salts are supposed to interact within a similar interaction mechanism with the other ash-forming matters.

To summarize the overall effect of the Ca-/Mg-based salts on the iron oxygen carriers in a visual manner, the samples were inspected after the experiments (Table 6). The inspection was based on the visually observable agglomeration behavior, and the effect was graded according to how easy or difficult it was to remove the samples from the crucibles after the experiments. It is known that some agglomeration can break down within the redox cycles,^59^ or the formed melt phase may only affect the other phases rather than the oxygen carriers.^49^ In our experiments, some samples were stuck to the crucible due to the interaction between alkali metal salt melts and the crucible material. Therefore, it was difficult to measure the agglomeration of the oxygen carriers without the possibility to measure the surface area of the particles. Among Ca-based compounds, CaCO_3_, Ca(NO_3_)_2_, and Ca_3_(PO_4_)_2_ showed the harshest agglomeration effect on the Fe oxygen carriers. When the surface area analysis was carried out, the smallest surface area was obtained for the sample consisting of Ca_3_(PO_4_)_2_ and Fe oxygen carriers. Based on the results, Ca-based compounds reduced the surface area of the oxygen carriers 13.3–50.6% from the surface area 4.21 m^2^/g of the pure Fe oxygen carriers. The results were similar for Mg-based compounds; however, MgCO_3_ showed a milder effect on the Fe oxides than CaCO_3_ did. Mg(NO_3_)_2_ and Mg_3_(PO_4_)_2_ showed the highest decrease of the surface area. Interactions between the Fe oxides and these salts reduced the surface area of the oxygen carriers by 48.2–59.4%.

**Table 6:** Summary of Visual Inspection/Surface Area of the Samples after Experiments

	effect^a^		surface area (m^2^/g)	
used compound in Fe oxides-ash-forming matter-based mixture	oxidation	reduction	oxidation	reduction
Ca(OH)_2_	3	4	3.54	3.65
CaCO_3_	2	1	3.09	2.23
CaCl_2_	2	2–3	3.16	3.08
Ca(NO_3_)_2_	1	2	2.11	2.97
Ca_3_(PO_4_)_2_	1	2	2.08	2.64
CaSO_4_	2	4	2.84	3.98
Mg(OH)_2_	4	4	3.68	3.60
MgCO_3_	2	3	3.16	3.22
MgCl_2_	3	4	3.51	3.78
Mg(NO_3_)_2_	2	1	2.18	1.71
Mg_3_(PO_4_)_2_	2	1	2.09	1.99
MgSO_4_	2	2–3	3.03	2.84
Ca-based synthetic ash	0	1	0.41^b^	0.67
Mg-based synthetic ash	0	0	0.38^b^	0.42^b^

a 1: High amount of coarse agglomerates. 2: Low amount of coarse agglomerates. 3: High amount of small agglomerates. 4: Low amount of small agglomerates. 0: Partially sintered.

b A representative sample was taken from the partially sintered body for BET analysis.

When the agglomeration effect of Ca- or Mg-based synthetic ashes was investigated, the ash consisting of Mg-based salts was found to be more aggressive than that containing Ca-based salts. From this point of view, a biomass containing a large amount of Mg, N, and P such as algae or some agricultural residues may not be the best candidates to be used as a fuel, where the Fe-based oxides will be used as the oxygen carriers. It is worth noting that a biomass containing a large amount of Ca could also create problems, since the reactivity of the oxygen carriers may decrease due to the formation of Ca–Fe-based solid compounds. However, some of the Ca–Fe-based reaction products, particularly Ca_2_Fe_2_O_5_, have been reported to be promising oxygen carriers for fuel conversion,^35^ so the formation of new compounds may also be positive.

## 3 Conclusions

In the study, the interaction between Fe oxides and alkaline-earth-based ash-forming matters was investigated to distinguish the different effects of Ca- and Mg-based salts. Iron oxide, one of the most used oxygen carriers, was used as an oxygen carrier, and a biomass composition was targeted to simulate the biomass-derived ash. It is vital to understand the nature of interaction between the ash-forming matters and oxygen carriers, since ash-forming matters may result in the deactivation of the oxygen carriers. There are a lot of studies focused on this interaction; however, these are mostly related to the alkali-metal-based ash-forming matters, since the alkali metal compounds can cause serious agglomeration issues due to the silicate-based formations. Alkaline earth species can also contribute to complex silicate phase formations, such as calcium or magnesium silicates. Moreover, recent studies reported that Ca- and Mg-based species may have different impacts on the oxygen carriers, even though they are supposed to have a similar chemical nature. This study presents only the effect of Ca/Mg-based ash-forming matters on the Fe oxides. Thermodynamic calculations were also carried out to reveal the possibly formed phases under the equilibrium conditions. The resulting phases provided by the calculations were consistent with those found in the experiments. This consistency is very important when an oxygen carrier would be chosen for a defined type of fuel, as the ash composition of fuels varies. The most significant result of the study was the formation of Ca–Fe-based oxides, when the synthetic ash consisting of Ca-based salts was used in the experiments. However, no formation of salt consisting of Fe and Mg oxides was observed, where the Mg-based salts were used as ash-forming matters in the experiments. The harshest salts in terms of the agglomeration were carbonates, nitrates, and phosphates. Their one-to-one interaction with the oxygen carriers resulted in serious agglomerations. In addition to this, the use of Mg-based salts in the synthetic ash caused more serious agglomeration than the use of Ca-based salts.

## 4 Materials and Methods

The starting materials were provided by Alfa Aesar as pure Fe_2_O_3_ and Fe_3_O_4_. Ash-forming matter representatives (Ca and Mg compounds) were all provided by Sigma-Aldrich. The iron-based oxygen carriers were prepared by the wet granulation technique to provide particle size distributions that are normal for oxygen carrier materials. The prepared oxygen carrier particles were then sieved to the size range of 125–180 μm. Hydroxides, chlorides, carbonates, nitrates, phosphates, and sulfates of Ca and Mg were used to represent ash-forming matters from the fuel. The mixtures consisting of Fe oxygen carriers and alkaline earth compound representatives were prepared to reveal the one-to-one interactions between the oxygen carriers and the model ash compounds. A previous experiment was taken as a reference to decide the composition of the mixtures.^60^ The chosen mixture was 90 wt % Fe oxide and 10 wt % alkaline earth compound. In addition, the oxides commonly present in biomass-derived ash in combination with Ca- or Mg-based salts were used to prepare two synthetic biomass ash compositions (see Table 7). To investigate the overall effect of these “synthetic biomass ashes” in a CLC process, iron oxygen carriers were mixed with the chosen synthetic ash in a weight ratio of 1 g of synthetic ash (Table 7) and 1 g of oxygen carrier, and the mixtures are exposed to a realistic reaction environment.

**Table 7:** Chemical Composition of the Synthetic Ashes

compound	amount (wt %)
SiO_2_	31
combination of Ca-a or Mg-based salts^b^	36
KOH	12.5
NaOH	12.5
Al_2_O_3_	4
Fe_2_O_3_	2
MnO	1
TiO_2_	1

a The mixture consists of an equivalent amount (∼7.2 wt % of each) of CaCO_3_, CaCl_2_, Ca(NO_3_)_2_, Ca_3_(PO_4_)_2_, and CaSO_4_.

b The mixture consists of an equivalent amount (∼7.2 wt % of each) of MgCO_3_, MgCl_2_, Mg(NO_3_)_2_, Mg_3_(PO_4_)_2_, and MgSO_4_.

Despite the fact that the ratio of the oxygen carrier to fuel-derived ash is higher in a real application, the mixture was prepared with a higher ash amount to represent the worst-case scenario for the oxygen carrier. Åbo Chemical Fractionation Database and the related studies were taken as a reference to determine the composition of the salt representatives in the synthetic ashes.^15,33^ The chemicals were mixed in an agate mortar with a pestle. To obtain a homogeneous mixture, acetone was used as a dispersing agent and the materials were mixed until a dry mixture was obtained.

The oxidation experiments took place in a tube furnace where an air atmosphere was used to simulate the air reactor, and a tube furnace was also used for the reduction experiments with reducing atmosphere consisting of 5 vol % H_2_ in Ar together with steam (50 vol %). The mixtures consisting of the oxygen carriers (Fe_2_O_3_ for reduction experiments and Fe_3_O_4_ for oxidation experiments) and one of the alkaline earth compound representatives were inserted in an alumina crucible, and heat treatments were carried out at 950 °C for 5 h. The same procedure was also performed for the synthetic ash/oxygen carrier mixtures. The gas flow rate was applied as 100 mL/min, and the heating/cooling rate was set to 10 °C/min in each experiment.

The samples obtained after each experiment were analyzed by X-ray diffraction (XRD) for identification of crystalline compounds. A Bruker D8 diffractometer was used with the characteristic Cu Kα radiation and settings 40 kV, 40 mA to collect diffraction data in the 2θ range of 15–80° with a step size of 0.01. Elemental mapping by scanning electron microscopy (SEM)–energy-dispersive X-ray spectroscopy (EDX) (JEOL JSM-7800F Prime) was also used to localize Ca and Mg compounds in the sample particles. Cross sections of the agglomerated particles were investigated via elemental mapping to reveal the agglomeration formation mechanism. To be able to see the cross section, the particles were embedded in an epoxy mold and the surface of the epoxy was polished to create a cross section. The specific surface area, as measured by BET-Micromeritics TriStar 3000, along with visual inspection of the mixtures after the experiments was also used to determine the extent of agglomeration.

FactSage 7.2-Equilibrium/Reaction Modules were used to evaluate the thermodynamic equilibria. The calculations were performed for 950 °C, and Pure Substance (FactPS)-Oxide (FToxid) databases were used under the presumption of an isothermal and standard state. The Gibbs energy minimization method was applied for the equilibrium module calculations. Calculated gaseous species present at less than 0.001 mol were ignored for the sake of simplicity. When the TEC results were reported in this study, only the phases that were present in amounts higher than 2.5 wt % in the resulting mixture were taken into consideration for both the sake of clarity and simplicity. This also helped us to compare the TEC with the experimental results.
